# Foundation species enhance food web complexity through non-trophic facilitation

**DOI:** 10.1371/journal.pone.0199152

**Published:** 2018-08-31

**Authors:** Annieke C. W. Borst, Wilco C. E. P. Verberk, Christine Angelini, Jildou Schotanus, Jan-Willem Wolters, Marjolijn J. A. Christianen, Els M. van der Zee, Marlous Derksen-Hooijberg, Tjisse van der Heide

**Affiliations:** 1 Institute of Water and Wetland Research, Radboud University Nijmegen, Nijmegen, the Netherlands; 2 Department of Environmental Engineering Sciences, University of Florida, Gainesville, Florida, United States of America; 3 Department of Marine Microbiology and Biogeochemistry, NIOZ Royal Netherlands Institute for Sea Research, Centre for Estuarine and Marine Ecology, Yerseke, the Netherlands; 4 HZ University of Applied Sciences, Vlissingen, the Netherlands; 5 Department of Biology, University of Antwerp, Wilrijk, Belgium; 6 Department of Aquatic Ecology and Water Quality Management, Wageningen University, Wageningen, the Netherlands; 7 Altenburg & Wymenga Ecological Research, Feanwâlden, the Netherlands; 8 Department of Coastal Systems, NIOZ Royal Netherlands Institute for Sea Research, and Utrecht University, Den Burg (Texel), The Netherlands; 9 Groningen Institute for Evolutionary Life Sciences (GELIFES), University of Groningen, Groningen, The Netherlands; Swedish University of Agricultural Sciences and Swedish Institute for the Marine Environment, University of Gothenburg, SWEDEN

## Abstract

Food webs are an integral part of every ecosystem on the planet, yet understanding the mechanisms shaping these complex networks remains a major challenge. Recently, several studies suggested that non-trophic species interactions such as habitat modification and mutualisms can be important determinants of food web structure. However, it remains unclear whether these findings generalize across ecosystems, and whether non-trophic interactions affect food webs randomly, or affect specific trophic levels or functional groups. Here, we combine analyses of 58 food webs from seven terrestrial, freshwater and coastal systems to test (1) the general hypothesis that non-trophic facilitation by habitat-forming foundation species enhances food web complexity, and (2) whether these enhancements have either random or targeted effects on particular trophic levels, functional groups, and linkages throughout the food web. Our empirical results demonstrate that foundation species consistently enhance food web complexity in all seven ecosystems. Further analyses reveal that 15 out of 19 food web properties can be well-approximated by assuming that foundation species randomly facilitate species throughout the trophic network. However, basal species are less strongly, and carnivores are more strongly facilitated in foundation species' food webs than predicted based on random facilitation, resulting in a higher mean trophic level and a longer average chain length. Overall, we conclude that foundation species strongly enhance food web complexity through non-trophic facilitation of species across the entire trophic network. We therefore suggest that the structure and stability of food webs often depends critically on non-trophic facilitation by foundation species.

## Introduction

Food webs and the feeding interactions they consist of have long been the focus of studies aiming to understand the complexity and stability of ecological communities [1]. There is a long tradition of studying individual consumer-resource (or ‘trophic’) interactions across the different species that make up a food web and describing the structure of these trophic networks. Collectively, this work has demonstrated that the properties of the trophic network itself, such as the number of species and links, connectance (realized fraction of all possible links), compartmentalization (also referred to as modularity), and the strength of trophic interactions, are important determinants of overall food web stability and robustness [2–7]. Furthermore, by extension, these findings indicate that changes in individual trophic interactions have the potential to cascade through the network, thereby destabilizing the entire food web and the corresponding ecosystem [8, 9].

Although food webs (i.e. trophic networks) are intensively studied paradigmatic examples of ecological networks [10, 11], organisms do not only interact through feeding interactions. Non-trophic interactions such as habitat modification, mutualism or competition for space have been suggested to indirectly affect food web topology and trophic dynamics by affecting the species in the network and the strength of trophic links [12–14]. Although numerous recent theoretical studies have therefore emphasized the urgency to integrate trophic and non-trophic interactions in ecological network analyses [14–19], empirical studies that do so remain scarce. Indeed, the few empirical studies that did address this knowledge gap suggest that food web structure (i.e. network topology) can be strongly influenced by species interactions outside the trophic network [12, 13, 20]. However, as these studies only include coastal systems and their number is very limited, it remains unclear to what extent these findings can be generalized across ecosystems. Moreover, whether non-trophic interactions typically affect specific species, trophic levels, or functional groups within the food web, or, alternatively, indiscriminately mediate species and their trophic interactions throughout the network has yet to be resolved. While multiple studies suggest that sessile species with a generally low trophic level benefit more than others from non-trophic facilitation [17, 21], other work suggests that facilitation also benefits higher trophic levels and more mobile species [20, 22, 23].

In this study, we test the general hypothesis that foundation species—spatially dominant habitat-structuring organisms (see e.g. [24–26])–modify food webs by enhancing their size (indicated by species number) and complexity (indicated by link density) via facilitation of species, regardless of ecosystem type. Additionally, we test that any change in food web properties caused by foundation species occurs via random facilitation of species throughout the entire food web or via targeted facilitation of specific species that belong to certain trophic levels or functional groups. Although foundation species are part of the food web like any other species (e.g. as prey or predator), numerous studies have shown that they strongly facilitate the associated community by creating new habitat and alleviating physical stress [12, 13, 21–23, 27–30]. This form of non-trophic facilitation by foundation species has been found to occur across a wide range of ecosystems and environmental conditions [31, 32]. In harsh coastal zones, corals, kelps, mussels, oysters, seagrasses, mangroves, and salt marsh plants facilitate organisms by attenuating currents and waves, providing aboveground structure for shelter and attachment, concentrating nutrients, and/or reducing desiccation stress during low tide exposure [24, 32]. In more benign systems, foundation species such as the trees in a forest, shrubs and grasses in savannahs, and macrophytes in freshwater systems, have also been found to play a major habitat-structuring role [31–34]. Ultimately, all foundation species increase habitat complexity and availability, thereby partitioning and enhancing the niche space available to other species [31, 35].

**Fig 1:**
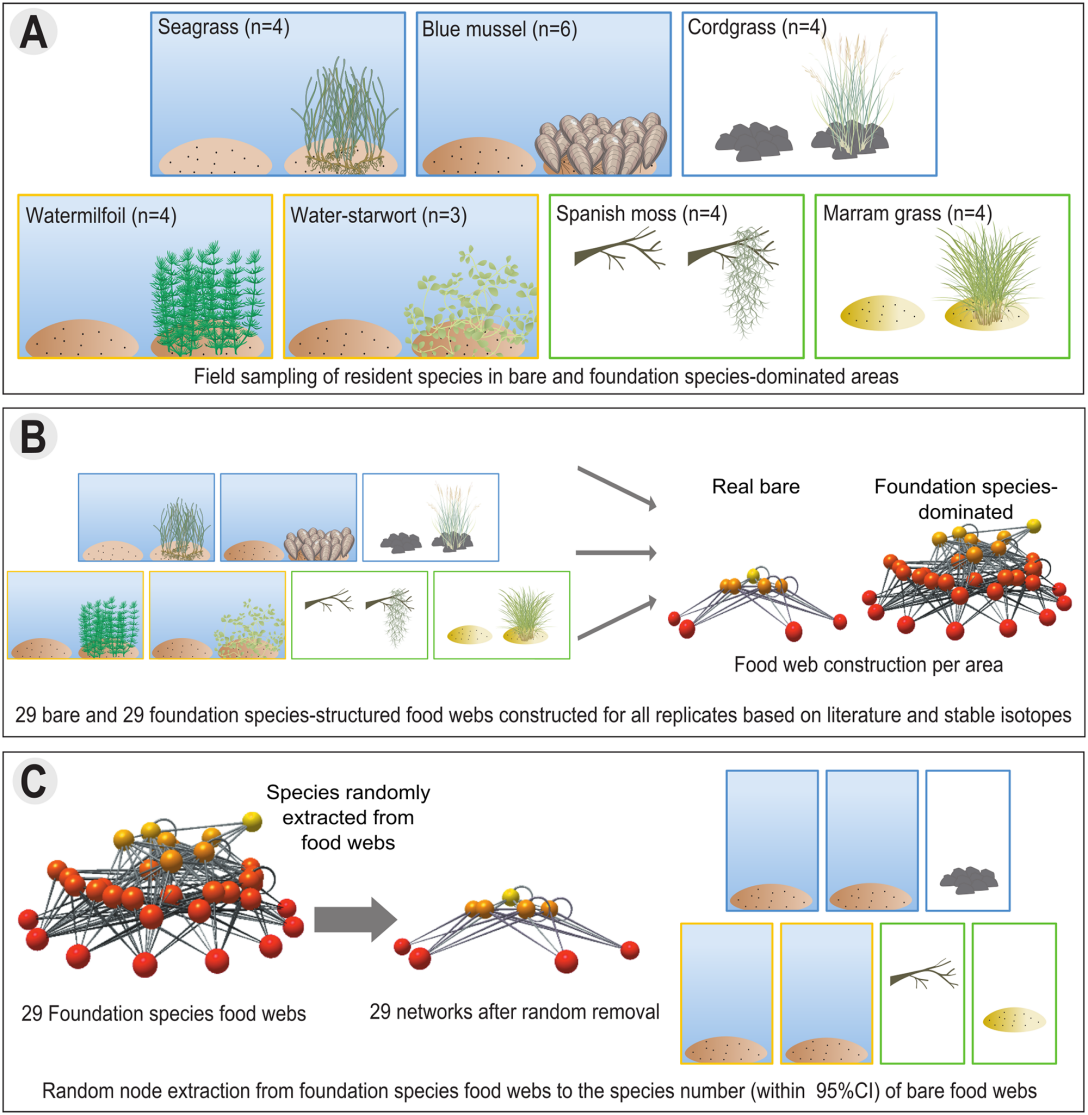
Illustration of the methods. (A) Seven ecosystems (including coastal (blue border), freshwater (yellow border) and terrestrial (green border)) were sampled (B) food webs were constructed, for both bare and foundation species-dominated replicate areas. (C) Finally, from each foundation species structured-food web we randomly removing nodes (i.e. species) until the species number matched the species number of the bare food webs within the 95% CI per ecosystem. Some of the symbols used in this figure were provided with the courtesy of Tracey Saxby, Dieter Tracey, Kim Kraeer and Lucy van Essen-Fishman, IAN Image Library (ian.umces.edu/imagelibrary/).

To test whether foundation species consistently increase food web size and complexity as hypothesized, we sampled species from areas dominated by foundation species with well-documented effects on their habitats (29 food webs) and from nearby bare, unmodified areas (29 food webs) in seven ecosystem types, including two terrestrial, two freshwater and three marine ecosystems. For each sampled area, we constructed the food web from the retrieved species using literature surveys, stable isotope analyses, and mixing models, after which we compared the properties of the bare and foundation species-dominated food webs. Next, we randomly removed species from each constructed foundation species-dominated food web to the number of species found in neighbouring bare areas (Fig 1). By comparing the properties of these simulated, random-removal networks to those of the real food webs, we investigate whether the observed food web modifications by foundation species arose from random or selective facilitation of trophic levels or functional groups across the trophic network.

## Results

**Fig 2:**
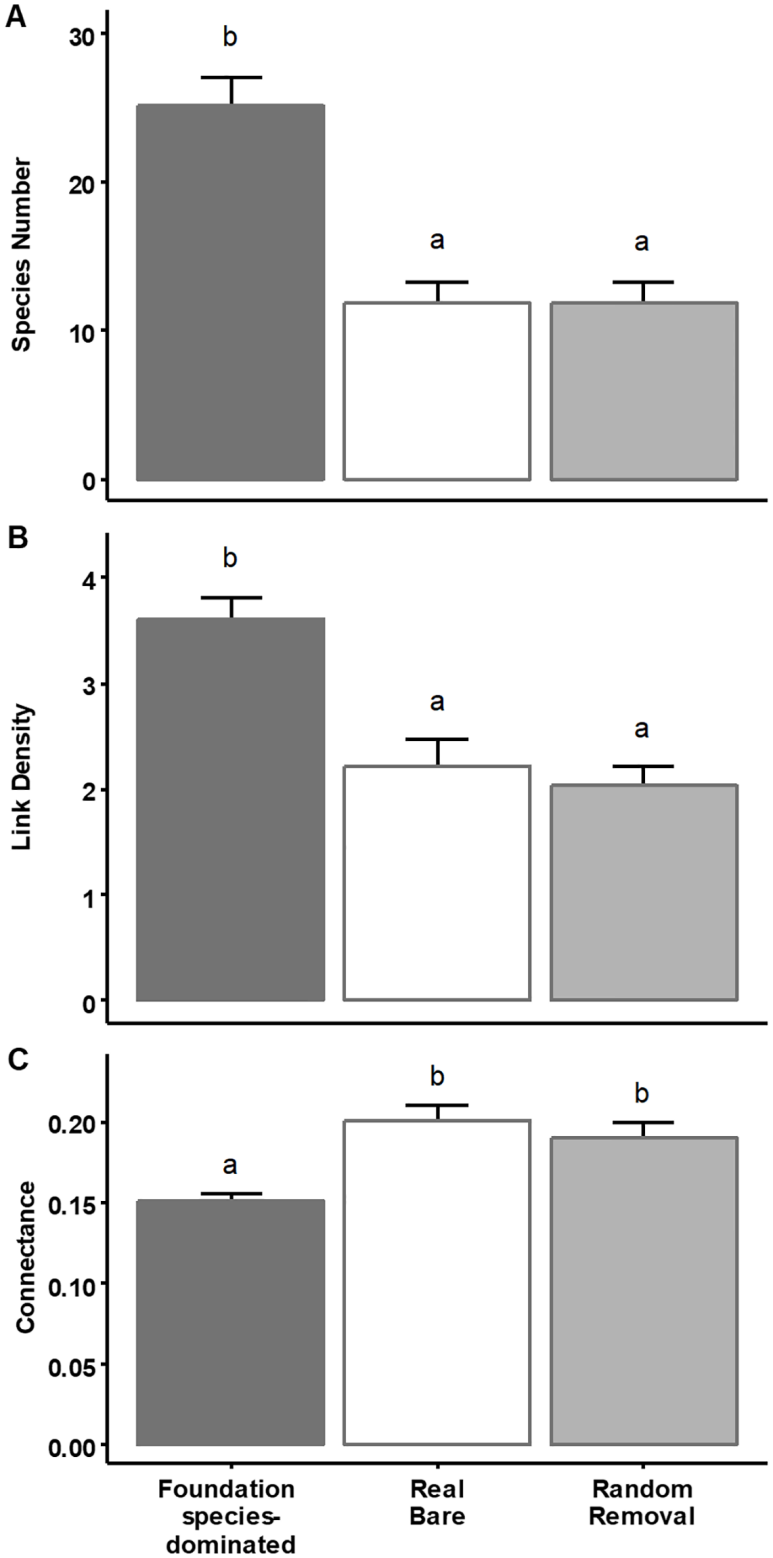
The presence of foundation species consistently changed food web properties (mean±SE) across ecosystems. Including (A) Species richness, (B) Link density, (C) Connectance. The random removal of nodes created networks which corresponded well with the properties of real bare food webs.

Despite large differences between all seven ecosystems in terms of environmental conditions and trophic network structure, we discovered that food web size and complexity, expressed as species richness and link density, respectively, were consistently enhanced in foundation species-dominated areas compared to food webs in nearby bare areas (Fig 2, Table 1 and S1 Fig). Specifically, species richness was on average 2.1 times higher, and link density increased 1.6 times in the presence of foundation species. Connectance—the realized fraction of all possible links—showed an opposite response and decreased 0.75 times. Out of the 19 food web metrics measured (full description of calculated metrics in S1 Table), 13 differed significantly between bare and foundation species-dominated areas, suggesting pervasive modifications to food web structure (Table 1). For instance, trophic distribution properties showed that the fraction of basal species decreased by 30%, the intermediate species fraction increased by 40%, and the top species fraction remained unchanged in response to foundation species presence. Furthermore, the average shortest chain length to a basal species increased by 30%, while the average trophic level and average path length between species (measure of energy transfer efficiency [36]) increased by 10%. Vulnerability—i.e. the number of consumers for each species—did not differ significantly for foundation species compared to other species, indicating that they are not consumed any more or any less compared to other species in the network (S2A Fig). We also found that the total number of trophic links to foundation species was almost half compared to the average number of trophic links per species in the rest of the network (S2B Fig). Moreover, this effect remained even when comparing only the number of links of basal foundation species versus other basal species (S2C Fig).

**Table 1:** Changes in food web properties between foundation species-dominated food webs, food webs from a bare area and random removal networks, and the result summary from the LMEs.

Metrics	AVERAGE ± SE^a^			Statistics^b^			
	Foundation species-dominated (FS)	Real Bare (BA)	Random Removal networks (RR)	F	p	Posthoc (FS, BA, RR)	Ecosystem
Species Number	25 ± 1.9	12 ± 1.4	12 ± 1.4	80	***	b, a, a	***
Link Density	3.6 ± 0.21	2.2 ± 0.24	2 ± 0.18	58	***	b, a, a	***
Connectance	0.15 ± 0.005	0.2 ± 0.009	0.19 ± 0.01	20	***	a, b, b	***
Vulnerability	3.5 ± 0.2	2.1 ± 0.23	1.9 ± 0.18	62	***	b, a, a	***
Generality	3.6 ± 0.21	2.2 ± 0.24	2 ± 0.18	58	***	b, a, a	***
Links	6.9 ± 0.41	4.1 ± 0.46	3.7 ± 0.35	62	***	b, a, a	***
Top fraction	0.29 ± 0.018	0.31 ± 0.023	0.36 ± 0.021	3.5	*	a, ab, b	ns
Intermediate fraction	0.47 ± 0.02	0.34 ± 0.044	0.37 ± 0.024	5.7	**	b, a, a	***
Basal fraction	0.24 ± 0.019	0.35 ± 0.032	0.26 ± 0.024	13	***	a, b, a	***
Herbivore fraction	0.26 ± 0.033	0.28 ± 0.031	0.24 ± 0.029	1.1	ns	a, a, a	***
Omnivore fraction	0.26 ± 0.037	0.24 ± 0.04	0.21 ± 0.035	2.8	ns	a, a, a	***
Carnivore fraction	0.24 ± 0.027	0.14 ± 0.028	0.29 ± 0.03	18	***	b, a, b	***
Cannibal fraction	0.14 ± 0.015	0.15 ± 0.023	0.17 ± 0.02	1.1	ns	a, a, a	***
Chain Length	1 ± 0.043	0.79 ± 0.056	1 ± 0.049	19	***	b, a, b	***
Trophic Level	2.1 ± 0.06	1.9 ± 0.085	2 ± 0.069	17	***	b, a, b	***
Max. Similarity	0.68 ± 0.017	0.57 ± 0.046	0.52 ± 0.05	8.1	***	b, a, a	***
Clustering	0.29 ± 0.034	0.26 ± 0.047	0.25 ± 0.037	0.87	ns	a, a, a	***
Path Length	1.9 ± 0.023	1.7 ± 0.059	1.7 ± 0.048	10	***	b, a, a	***
Compartmentalization	0.23 ± 0.009	0.19 ± 0.019	0.2 ± 0.019	2.8	ns	a, a, a	***

^**a**^ Foundation species-dominated food webs (FS), Real Bare food webs (BA), Random removal networks (RR).

^b^ Effects were tested in a mixed model with the ecosystems as random factor.

***: p<0.001,

**:p<0.01,

*:p<0.05,

. :p<0.1,

ns: not significant

**Fig 3:**
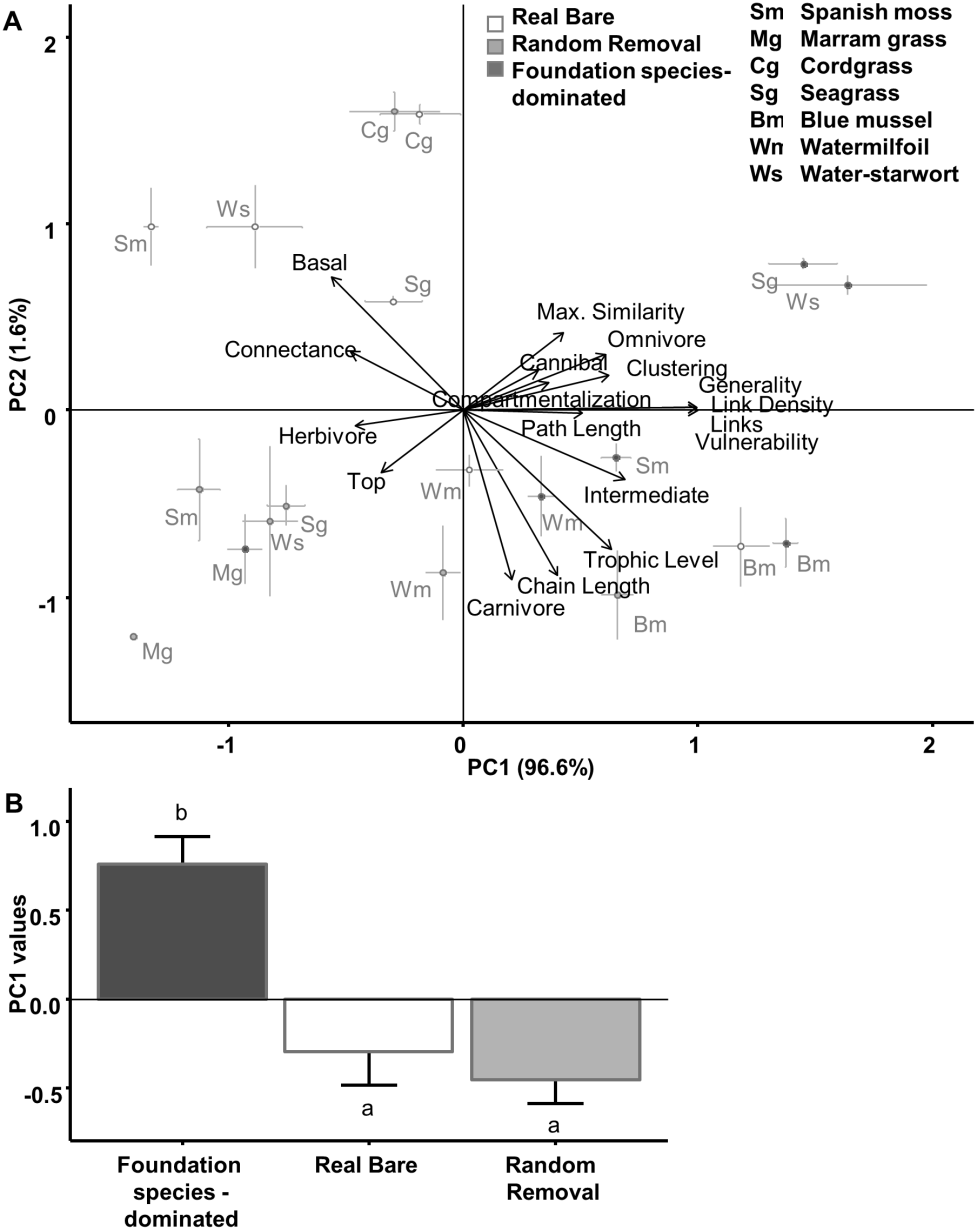
PCA Axis 1 clearly differentiated between bare and Foundation species-dominated, but not between bare and random removal networks. (A) Averaged PCA values (mean±SE) of all food web metrics describing both field and simulated food webs of foundation species-dominated and bare areas. Arrows are projected food web metrics (total variation 1090, axis 1: 96.6%, axis 2: 1.6%). (B) Scores of Principle Component axis 1 explained by real bare versus foundation species-dominated (p < 0.0001), and real bare versus random removal networks (ns).

Random removal of species from the foundation species-dominated food webs until the species number matched the numbers found in bare areas simplified trophic networks, and altered most food web properties such that they resembled those from the bare areas we sampled (Table 1 and Fig 2). Moreover, when we combined the food web metrics from foundation species-dominated, real bare, and random removal food webs in a Principle Components Analysis (PCA), we discovered that most of the variation (96.6%) in the metrics could be condensed onto a single Principle Component axis, PC1 (Fig 3, PC2 explained only another 1.6% of the variation). PC1 clearly differentiated real bare, and foundation species-structured food webs (F_2, 78_ = 79.9, p<0.0001), but did not differentiate between real bare and random removal food webs (Fig 3B). This indicates that, when analyzing the overall repsonse of food web metrics, networks created by random species removal corresponded well with those observed in the real bare areas.

Our PCA results are supported by more in-depth comparative analyses of individual metrics. Whereas 13 out of 19 metrics included in our analyses differed significantly between food webs from foundation-dominated and bare areas, only 4 out of 19 metrics deviated significantly when comparing real bare and random removal food webs (Table 1 and S1 Fig). Interestingly, these four metrics—the basal fraction, number of carnivores, average shortest chain length and average trophic level—also showed the strongest correlation with PC2 from the PCA (Fig 3A). Specifically, we found that, after randomly removing species from real, foundation species-dominated food webs, the fraction of basal species was significantly lower, while the number of carnivores, average chain length, and average tropic level were higher compared to real bare area food webs.

## Discussion

Multiple theoretical studies have suggested that habitat-modifying organisms (i.e. foundation species, ecosystem engineers) significantly enhance food web size (i.e. species richness) and complexity (i.e. link density) by affecting species and links through non-trophic interactions [14–17]. Although this hypothesis was recently supported by two empirical studies of coastal ecosystems [13, 20], it remained unclear whether these findings could be generalized to other ecosystems. By comparing foundation species-dominated habitats with nearby bare habitats where these foundation species ware absent, we demonstrate that their presence consistently increased food web size and complexity across seven terrestrial, freshwater and coastal ecosystems. We found that, in all seven investigated ecosystems, facilitation by foundation species increases the number of species, and the number of links per species, while decreasing link saturation (connectance). Moreover, our analyses provide support for the hypothesis that non-trophic facilitation by foundation species facilitates associated species throughout the trophic network.

As foundation species in our study were themselves also part of the food web, the observed changes in food web complexity and properties could theoretically have resulted from their trophic role instead of non-trophic facilitation. However, although the trophic network did increase in size in the presence of foundation species, the number of species feeding on foundation species—i.e. their vulnerability—did not differ significantly from the number of species feeding on other species in the network (S2A Fig). Moreover, we found that the number of trophic links to foundation species was in fact much lower compared to the average number of trophic links of other species in the network even when comparing only basal species (S2B and S2C Fig). Although foundation species with only few trophic links could theoretically still be important in the food web by serving as a vital food source for highly connected species, this is unlikely as a large number of studies have revealed that the palatability of foundation species in general can be considered rather low [13, 21, 37, 38]. These results suggest that trophic facilitation on its own is not a likely explanation for the observed enhancements of food web complexity by foundation species. Instead, non-trophic facilitation would seem to be the main driver, corroborating a large body of earlier work showing that habitat modification and stress amelioration by foundation species is critical to their enhancement of species richness [13, 20, 23, 24, 30, 32]. Thus, our empirical work provides compelling cross-ecosystem evidence for the hypothesis that non-trophic facilitation by foundation species, rather than their trophic role, can be an important driver of food web structure [12, 13, 15–17].

The underlying mechanisms by which foundation species non-trophically facilitate associated species may differ widely across ecosystems, including those we investigated. Simple provisioning of attachment substrate and three-dimensional structure has been found to be an important mechanism of facilitation by all foundation species [26, 31, 32, 39]. In addition, species may also be benefit from foundation species because the foundation species concentrates critical resources (e.g. water, detritus), or mitigates physical stress resulting from currents, waves, wind, sediment instability, drought or high temperatures [13, 20, 30, 40]. Furthermore, indirect facilitation through trophic pathways is also possible. For instance, foundation species may trap or accumulate nutrients, detritus, and other resources (S3 Fig), or mediate a trophic cascade in which predators depend on prey that is non-trophically facilitated by the foundation species [37, 41].

Our analyses reveal that random removal of species from foundation species-dominated food webs yielded food webs very similar to bare areas (Fig 3). Yet, despite their similarities, four metrics—the basal species fraction, carnivore fraction, average chain length, and average trophic level—deviated, suggesting that non-trophic random facilitation can explain the observed food web modifications to a large extent, but not completely. In foundation species-dominated food webs, the number of basal species is relatively lower, while the number of carnivores is higher than predicted based on random facilitation. Foundation species can compete for space with larger sessile species that occur at the base of the food web, possibly explaining the relatively reduced representation of basal species in foundation species-dominated food webs [12]. Furthermore, carnivores may be particularly dependent on habitats that support high densities of prey and which are characterized by low physical stress—conditions created by the foundation species [42, 43], resulting in their overrepresentation in foundation species-dominated food webs. As a result of the relatively higher number of carnivores that occur at higher trophic levels, the average chain length and average trophic level likely tends to be higher than expected from random facilitation in these conditions.

One important explanation for why foundation species facilitate other species that occur throughout the entire food web is that they provide three-dimensional structure [31], thereby enhancing niche availability and complexity (e.g. via the creation of an epi-benthic next to an endo-benthic community). Earlier work showed that random removal of species has a lower impact on food web structure compared to the removal of specific species or trophic groups. Specifically, studies by Sole and Montoya [44], Dunne *et al*.[4] and Mulder *et al*. [45] all found that random removal of species leads to less secondary extinctions and a higher robustness of food webs compared to a more targeted removal of species. Hence, based on this earlier work, our findings suggest that foundation species, by facilitating species across trophic levels, sustain food webs that are more robust than those that would be generated if they were to facilitate specific trophic levels, feeding guild or functional groups.

Although foundation species may in principle stabilize and enhance the size of food webs through niche creation, this does not necessarily mean that foundation species-supported food webs are more resilient to real-world disturbances. In the foundation species-supported food webs that we analyzed, over 50% of the species appear directly or indirectly dependent on non-trophic facilitation by foundation species (Table 1). This implies that the food webs associated with foundation species are likely very sensitive to disturbances that affect the health and functioning of foundation species. At present, many ecosystems worldwide that are structured by foundation species—including seagrass meadows, salt marshes, coral reefs, peatlands and rainforests—are declining at an alarming rate due to anthropogenic disturbances [46–50]. Our findings suggest that to preserve complex but stable food webs across ecosystems, it is vital to prioritize the conservation and restoration of the foundation species that support them.

## Methods

### Study systems

Foundation species are sessile, spatially dominant habitat-modifiers that create physical structures with their own body tissue. Many plants such as seagrasses or trees can be considered foundation species, but many corals and bivalves fit this definition [25, 26, 31, 32]. These species often facilitate their own growth in a scale dependent manner. This behavior results typically results in emergent pattern formation [51–53] with locally clustered areas of foundation species and adjacent bare habitat without foundation species (i.e. rocks or sand).

We sampled 58 food webs from seven different ecosystem types using a consistent methodological approach. Although the abiotic environmental conditions vary widely across these ecosystems, all are typified by the presence of a spatially dominant foundation species that enhances habitat complexity, and mitigates environmental stress [26, 31, 32]. Our study included three coastal ecosystems: (1) intertidal seagrass beds dominated by *Zostera noltii* in Banc d’Arguin, (Mauritania), (2) cordgrass-dominated (*Spartina alterniflora*) fringing marshes growing on the cobble beaches of Rhode Island (USA), and (3) intertidal blue mussel beds (*Mytilus edulis*) in the Wadden Sea (the Netherlands). Apart from enhancing habitat complexity, the foundation species of these coastal ecosystems all attenuate current and waves, stabilize the substrate, and provide shelter and attachment for other species [22, 54–56]. In addition, seagrass and mussels trap large quantities of suspended particles from the water column, whereas cordgrass provides shading and stabilization of cobble stones [57]. We also included two freshwater ecosystems: (1) Watermilfoil (*Myriophyllum alteniflorum*) dominated shallow lakes in a dune slacks with standing water on the Wadden Sea island of Terschelling (the Netherlands), (2) and Water-starwort (*Callitriche obtusangula*) dominated slow flowing streams (Desselse Nete, Belgium). Freshwater macrophytes have been found to facilitate other species by providing structure and shelter against flow stress and predation by fish and apex invertebrate predators [58–60]. Finally, we sampled two terrestrial systems: (1) marram grass-dominated (*Ammophila arenaria*) dunes at Terschelling, and (2) Spanish moss (*Tillandsia usneoides*)-dominated live oaks in Georgia (USA). Marram grass attenuates wind and heat stress (S4 Fig), while Spanish moss reduces desiccation and predation stress relative to adjacent stretches of bare live oak branch. [23]

### Sampling

To sample the food web in each foundation species-colonized and bare habitat, we followed the general methodology by Van der Zee et al (2016). Within each ecosystem we sampled two contrasting habitat types: areas dominated by foundation species and bare areas where the foundation species was absent. To minimize differences in underlying environmental conditions, all habitats were sampled in a pairwise manner, with the exception of Banc d’Arguin where this was not possible as habitat modification effects occur at much larger scales compared to the other ecosystems. Instead, to avoid dissimilarities in the underlying conditions due to environmental gradients, all sampling stations were selected based on a random spatial distribution, with similar elevation, distance to the gully, maximum fetch length and Exposure Index—an integrative measure of wave exposure (see [13]). A full description of the sampling strategy per ecosystem can be found in S1 Text in Supporting Information. In all seven systems we sampled 3 to 6 replicate areas (58 food webs total; Fig 1). For each sampled area, we collected and identified all abundant species that collectively represented at least 95% of the biomass in each habitat.

After collection, all samples were stored in the freezer (-20°C) until further analysis. The samples were then dried to constant weight, either by means of an oven set between 50 and 60 °C, or by means of freeze-drying. Dried samples were homogenized using a hand mill or mill grinder (Retsch, Aartselaar, Belgium). Finally all samples were analyzed in duplicate on an Isotope-ratio Mass Spectrometer (IRMS) (Thermo Scientific, Waltham, Massachusetts, USA) for *δ*^13^ carbon isotopic signal and *δ*^15^ nitrogen isotopic signal.

### Food web reconstruction

Dichotomous food webs were constructed for each sampled area (Fig 1A). We used scientific literature, databases and expert knowledge to construct a maximized theoretical network that included all possible trophic links for the sampled organisms. To include only ecologically relevant interactions and omit incidental ones, we then constrained each maximized network by removing rare species (i.e. <3 individuals counted in each ecosystem), and highly improbable interactions, for instance those based on size discrepancies between predators and prey based on expert judgement [12, 13]. Finally, based on diet reconstruction using stable isotope bi-plots of *δ*^13^C and *δ*^15^N and Bayesian mixing models (R-package SIAR [61]), we further constrained the network by removing improbable trophic links where prey contributed less than 5% to the consumer’s diet [13, 20]. Food webs were constructed in a dichotomous n*n matrix, in which the columns represent all n species as predators and the rows represent all n species as prey. Based on these matrices, we calculated a number of widely used food web metrics per replicate food web (Table 1).

### Trophic importance of foundation species

To determine the extent to which foundation species trophically affected food web structure, we assessed their trophic contribution relative to those of other species by comparing their vulnerability metric (Table 1) to the average vulnerability of all other species in the food web. High values would indicate that the foundation species is a relatively important food source in the network and may thus strongly affect food web properties via its trophic role. We also determined the number of links of foundation species, and compared it to the average of all other species in the food web (‘Links’ in Table 1). In addition, as most foundation species are basal species, which do not feed on other species in the food web and may therefore have fewer overall links, we also compared the number of links of basal foundation species to the average number of links of other basal species in the food web.

### Random species removal

To test whether observed food web modifications by foundation species arose from random or selective non-trophic facilitation of species, links or levels across the trophic network, we randomly removed species from foundation species-dominated food webs to match the number observed in neighbouring bare systems. Specifically, we pruned the foundation species-structured food webs by randomly removing species, also deleting species that became trophically isolated from all other species (i.e. no remaining feeding links or not connected to any other species) as a result of this random removal procedure. For each food web, we randomly removed nodes until the number of species in the remaining trophic matrix fell within the 95% confidence interval of the average number of species in the corresponding bare plots (Fig 1C). Finally, we calculated all abovementioned food web metrics (Table 1) again for the randomly-constrained model-derived ‘bare’ food webs. If foundation species facilitate food webs in a random fashion (i.e. the foundation species does not have disproportionate effects on specific species, trophic levels or functional groups), the empirical bare food webs and their properties should be indistinguishable from those of the simulated bare plots after our random pruning procedure.

### Statistical analyses

We used Linear Mixed-Effect models (LME) to compare individual food web metrics (listed in Table 1) between food webs from foundation species-dominated areas, bare areas and the random removal procedure (hereafter food web type) Significance was tested using one-way ANOVA F-tests with a Satterthwaite approximation of the degrees of freedom (package lmerTest in R[62]), and Tukey posthoc tests to differentiate between food webs type. In these analyses, we used food web type as a fixed factor and ecosystem as a random factor.

Trophic dependency (i.e. average vulnerability) and total number of links of the foundation species versus the average number of links of the other species in that network were analyzed using foundation species or ‘other species’ as fixed factor and ecosystem as random variable in a LME and an ANOVA F-test. Residuals of all models were checked for normality by Shapiro Wilk’s test and a qq-plot and response variables were log-transformed when necessary. Trophic dependency on basal foundation species versus the average number of links of other basal species was analyzed in the same way.

To assess the overall combined response of food web metrics to food web type, we constructed Principal Component analysis (PCA), analyzing the different replicate food webs as samples and food web metrics as variables. Scores of the first Principle Component axis were taken as a composite measure of food web structure and were analyzed with a LME model and tested with a one-way ANOVA F-test using food web type as a fixed factor and ecosystem as random variable to assess the effect of our random removal procedure on overall food web structure.

## Supporting information

S1 Fig Food web properties averaged and per ecosystem. Properties are arranged in Marine, Freshwater and Terrestrial (Sg: Seagrass, Cg: Cordgrass, Bm: Blue mussel, Wm: Watermilfoil, Ws: Water-starwort, Mg: Marram grass, Sm: Spanish moss) averaged for Foundation species-dominated food webs (FS), food webs from bare areas (BA) and random removal networks (RR).
(TIF)

S2 Fig Trophic dependency on foundation species compared to other species in the network. Trophic dependency is not higher for foundation species when expressed as vulnerability (A) the total number of links compared to the other species (B), or the number of outgoing links of basal foundation species versus other basal species (C)) for which the number of links equals vulnerability.
(TIF)

S3 Fig Watermilfoil indirectly stimulates the food web by increasing periphyton availability. (A) Watermilfoil increased periphyton cover. (B) Trophic dependency on periphyton as a food source is much higher than on Watermilfoil.
(TIF)

S4 Fig Stress mitigation in marram grass plots compared to bare sand. Marram grass mitigates (A) wind speed and (B) maximum temperature and temperature variability.
(TIF)

S1 Table Food web metrics calculated for all food webs. (XLSX)

S1 Text Detailed description of sampling methods per model ecosystem. (DOCX)
