# Supplementary figures and images for: Foundation species enhance food web complexity through non-trophic facilitation
Source: PLoS One. 2018 Aug 31;13(8):e0199152. doi: 10.1371/journal.pone.0199152 (PMC6118353; doi:10.1371/journal.pone.0199152)

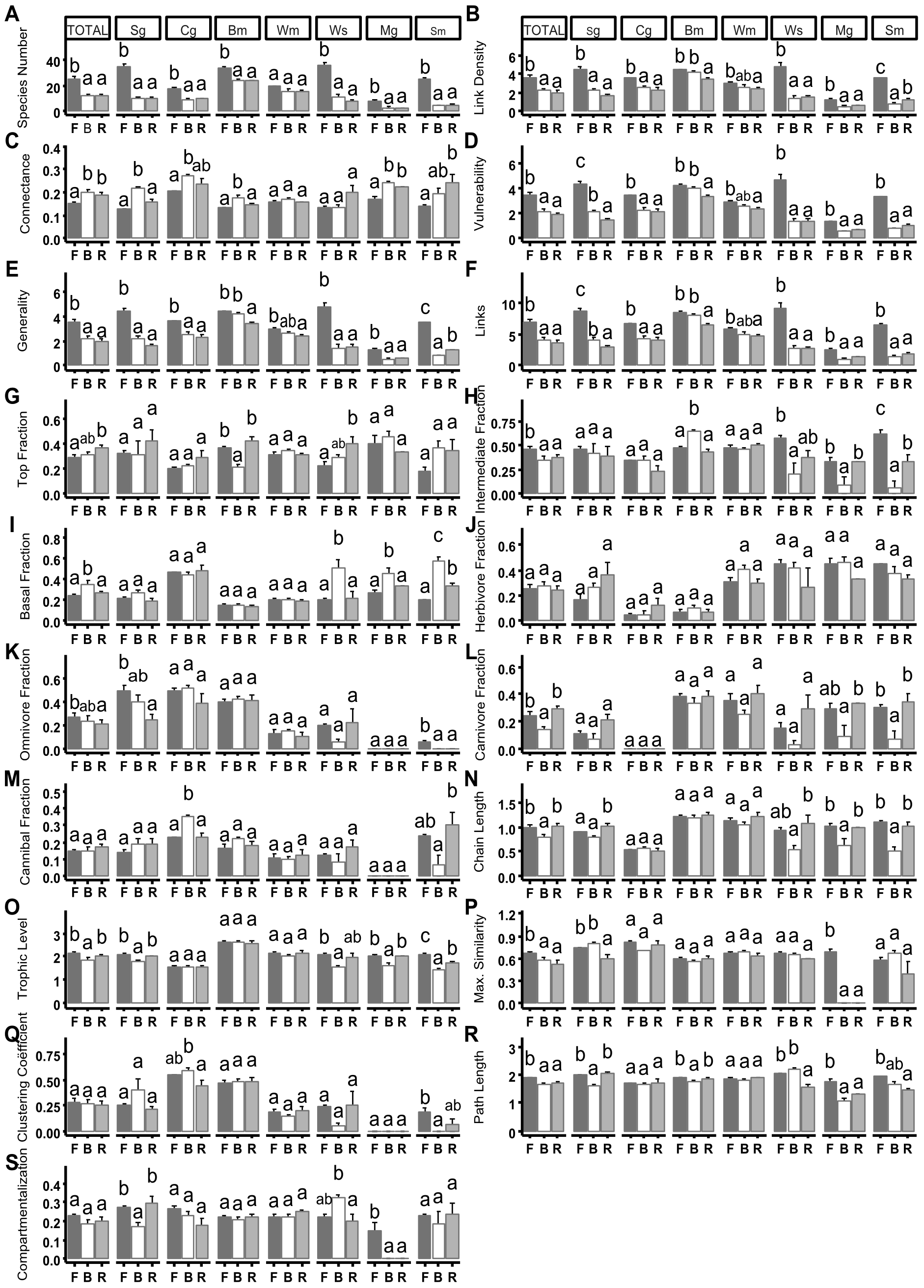

Supplement: S1 Fig — Properties are arranged in Marine, Freshwater and Terrestrial (Sg: Seagrass, Cg: Cordgrass, Bm: Blue mussel, Wm: Watermilfoil, Ws: Water-starwort, Mg: Marram grass, Sm: Spanish moss) averaged for Foundation species-dominated food webs (FS), food webs from bare areas (BA) and random removal networks (RR). (TIF) [file pone.0199152.s001.tif]

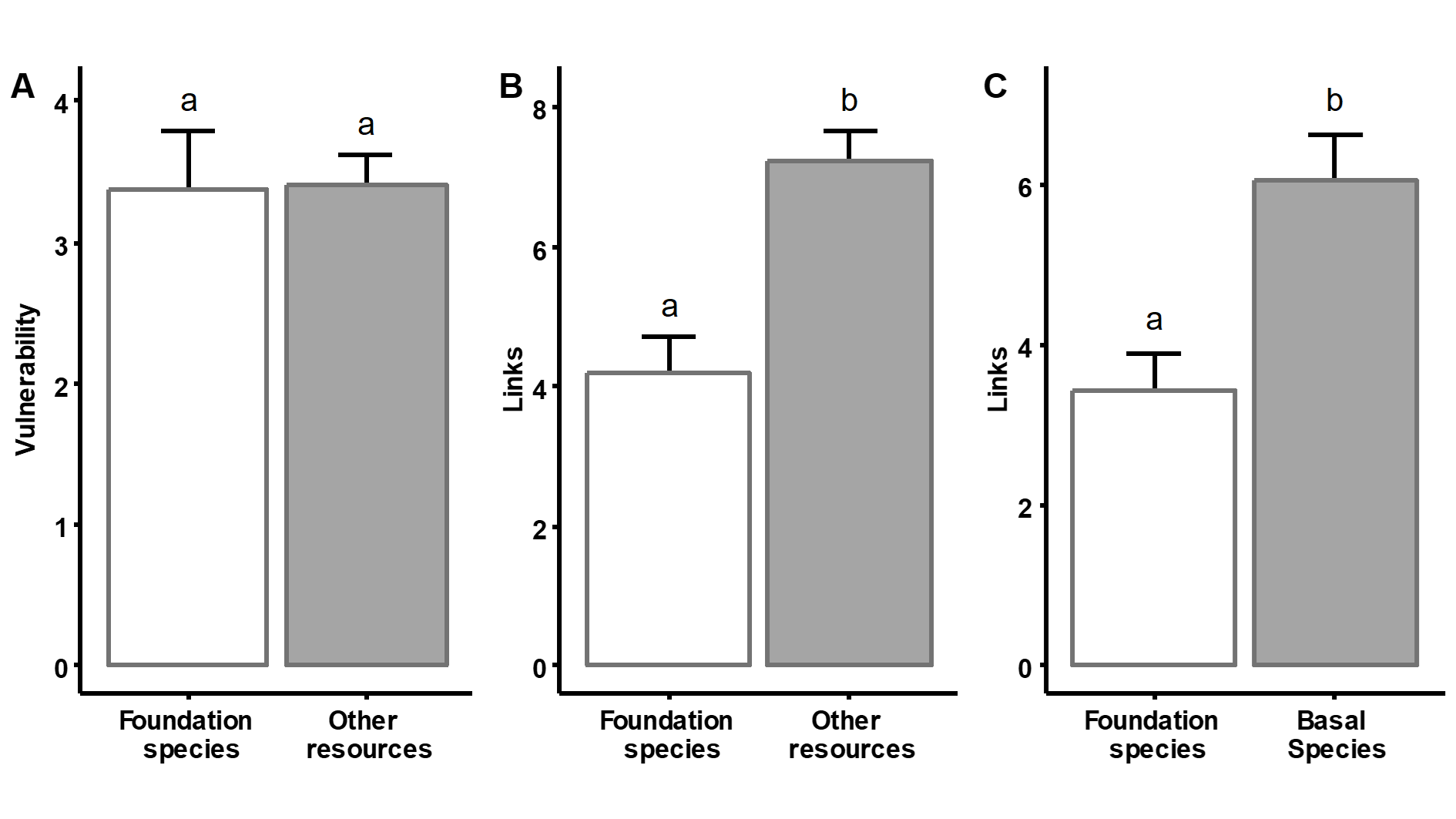

Supplement: S2 Fig — Trophic dependency is not higher for foundation species when expressed as vulnerability (A) the total number of links compared to the other species (B), or the number of outgoing links of basal foundation species versus other basal species (C)) for which the number of links equals vulnerability. (TIF) [file pone.0199152.s002.tif]

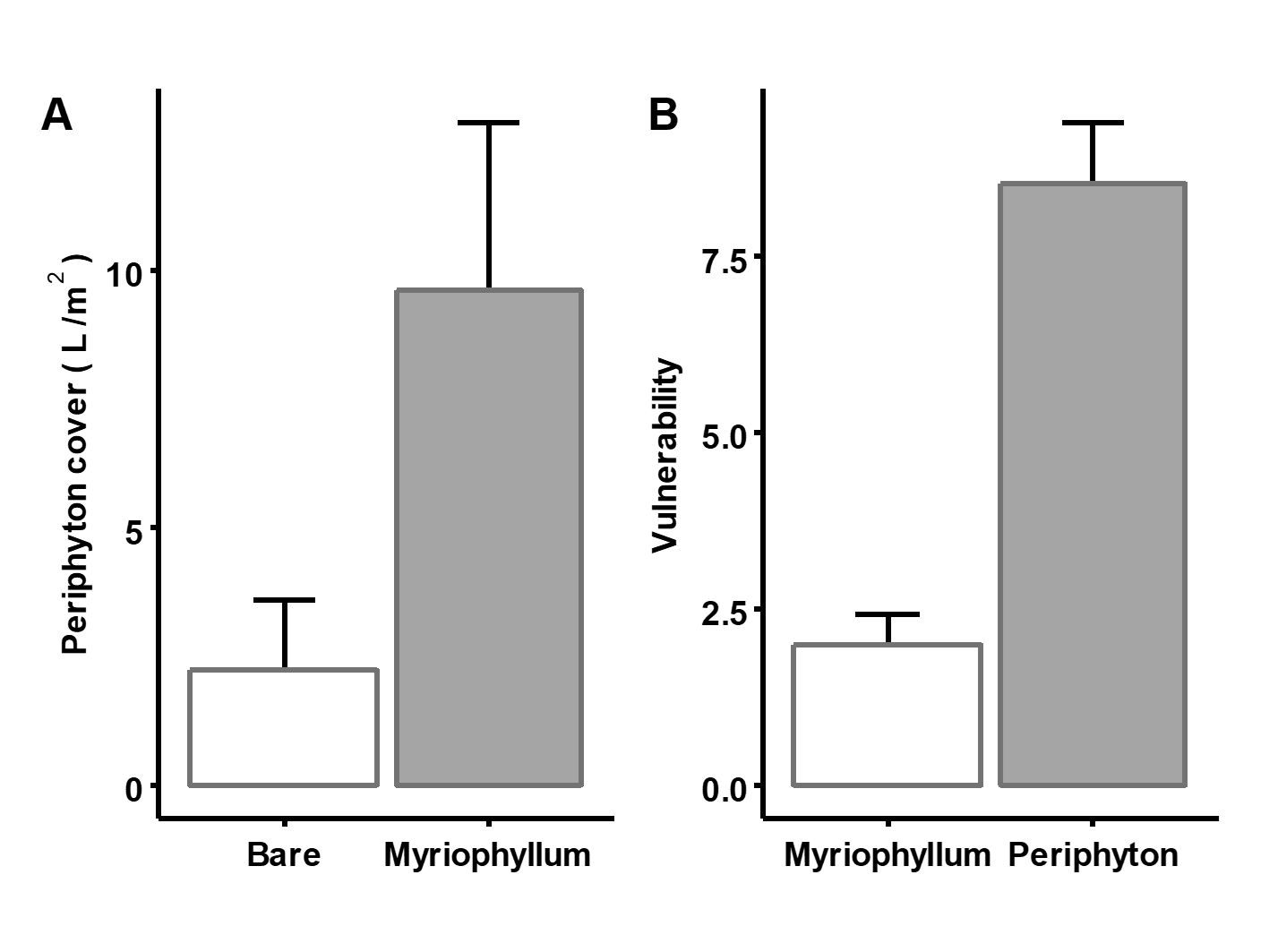

Supplement: S3 Fig — (A) Watermilfoil increased periphyton cover. (B) Trophic dependency on periphyton as a food source is much higher than on Watermilfoil. (TIF) [file pone.0199152.s003.tif]

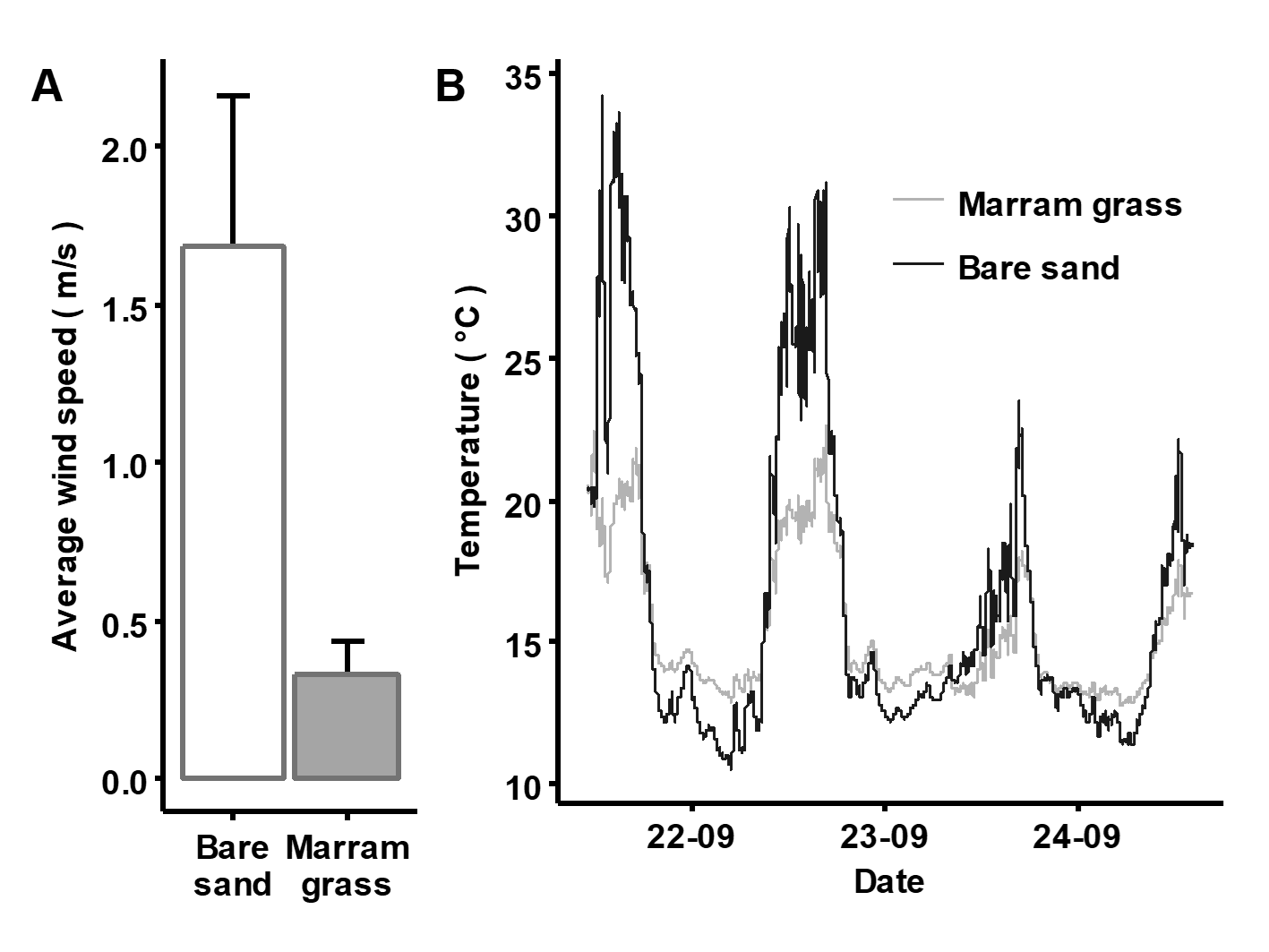

Supplement: S4 Fig — Marram grass mitigates (A) wind speed and (B) maximum temperature and temperature variability. (TIF) [file pone.0199152.s004.tif]
