# Supplementary material for: Foundation species enhance food web complexity through non-trophic facilitation
Source: PLoS One. 2018 Aug 31;13(8):e0199152. doi: 10.1371/journal.pone.0199152 (PMC6118353; doi:10.1371/journal.pone.0199152)
Supplement: S1 Text — (DOCX) [file pone.0199152.s006.docx]

Supplementary Materials

**Materials and Methods: Detailed description of sampling methods per model ecosystem.**

Fringing marshes, New England, USA

We randomly selected four replicate plots with similar elevation, distance to the gully, maximum fetch length and Exposure Index at low tide for both bare cobble stone and *Spartina alterniflora*. Within each plot, a 25×25 quadrat was placed to count the number and abundance of resident (i.e., not migrating with the tide) species.

Seagrass meadows, Banc d’Arguin

We established four plots of bare sand and seagrass meadows in 50-m diameter circles during low tide. Within this circle, 4 replicate areas for sediment and (endo)benthos samples were selected. An extensive description of the sampling of both the cord grass and the seagrass can be found in van der Zee et al. (13)

#### Watermilfoil in a dune lake

In dune lake El Dorado on Terschelling Island, The Netherlands (53°24'N, 5°16'E), we selected bare, sandy patches and patches where Watermilfoil (*Myriophyllum alterniflorum*) covers up to a 100% of the area. We randomly selected 4 replicate plots for each type of habitat and sealed the fauna present in the plot by placing a sampling ring with a diameter of 45 cm. The fauna in the water layer was sampled by sieving all the water within the ring over a 1 mm mesh sieve. The periphyton layer on and sediment samples was taken using a 50 ml-corer. All plant material in the plot was harvested and macrofauna hiding between the plants were collected later in the lab. Zooplankton and floating algae samples were collected separately for the whole lake. To this end, surface water was filtered over a 200-µm zooplankton net and finally precipitated onto Whatman GF/F filters after which it could be collected by scraping the filter carefully. Zooplankton was collected using a zooplankton net, and subsequently filtered onto Whatman GF/F filters after which it was collected by scraping the filter. All samples were stored at -20˚C until further processing. Fauna species were identified in the lab to the lowest feasible level, mostly to family level. All samples were then rinsed with demineralized water, freeze-dried and homogenized using a ball grinder (Retch). Homogenized samples were weighed in tin cups and analyzed for carbon and nitrogen stable isotope composition on an isotope ratio mass spectrometer (IRMS, Thermo Scientific).

#### Marram grass in the dunes of Terschelling, The Netherlands

In the dunes of the Terschelling Island in The Netherlands (53°25'N, 5°24'E), we randomly selected 4 replicate plots of bare, sand dune habitat and within the marram grass tussocks (*Ammophila arenaria*), which were covered up to 90% with marram grass. All plots were sampled on the same dune, no more than 50 meters apart.

We sampled flying insects in the plots by sweeping a fly net back and forth fifteen times. Then we manually collected the ground dwelling fauna by placing a sampling ring with a diameter of 45 cm around the plot. We collected all marram grass present in a plastic bag which was later sorted for fauna hiding within the rolled up of the grass.

A sample of 5 liter taken from the sediment up to 10 cm deep and put through a 5 mm mesh sieve. Also 1 liter from this 5 liter was sieved at a mesh size of 1 mm. No subterrestrial species were found in these samples. Invertebrates were then identified, mostly to species level. All samples were dried at 60°C until stable weight and homogenized using a ball grinder (Retch). Homogenized samples were weighed in tin cups and analyzed for carbon and nitrogen stable isotope composition on an isotope ratio mass spectrometer (IRMS, Thermo Scientific).

#### Spanish moss at Sapelo Island, Georgia USA

Spanish moss food web samples were collected on Sapelo Island, Georgia USA (31°25'N 81°16'W). In 4 trees of Southern Live oak (*Quercus virginiana*), we randomly sampled the food webs of both 8 paired replicate ~50 cm long bare branches and branches with a large ‘festoon’ of Spanish moss (*Tillandsia usnoides*). The 8 collected samples were pooled to construct one bare food web and one Spanish moss food web per Live oak.

An airtight bag was used to enclose all invertebrates in a volume of ~40 cm^3^ around the branch. Mobile species were brushed off the branch in the bag with a stiff brush. All invertebrates was then extracted from the bag with a suction sampling method, using an insect screen attached to suction device (i.e. leaf blower). Invertebrates where stored in at -20˚C until identification and Stable Isotope Analysis. Species were identified in the lab to the lowest feasible level, mostly family. Likely food sources like fungi and bark were collected separately to determine stable isotope signature. All samples were dried at 60°C until stable weight and homogenized using a ball mill (Retch). Homogenized samples were weighed in tin cups and analyzed for carbon and nitrogen stable isotope composition on an isotope ratio mass spectrometer (IRMS, Thermo Scientific).

#### Intertidal Blue mussel beds at the wadden sea, The Netherlands

We sampled and reconstructed the food webs of 6 intertidal mussel bed and adjacent bare mudflat in a paired design across the Dutch wadden sea. All fauna was identified to species level. An extensive overview of the methods used to collect this food web is given in the paper of Christianen et al (19).

#### Water-starwort in Desselse Nete, Belgium

Aquatic macroinvertebrates were collected from 3 monospecific Water-starwort (*Callitriche obtusangula*) patches and neighbouring bare patches in the Desselse Nete, a sand bottom lowland stream in the north of Belgium (51°14'53" N, 5°4'53" E). In the studied reach, stream width varied between 3.5 and 5.5 m with an average depth of 58 cm and an average discharge was 0.69 m^3^s^-1^. Macroinvertebrates were collected both on the vegetation and in the sediment. Within macrophyte stands, macroinvertebrates were collected using a cylindrical box-sampler (total volume: 6663 cm3). Macroinvertebrates in the sediment were collected inside each vegetation patch and in neighbouring bare patches. For each sediment sample three sediment cores were collected and pooled using a core sampler (diameter 5.4 cm), resulting in a total sediment area of 63.7 cm2 for each sample. Additional food web components, including organic matter, macrophytes and epiphyton, were also collected. Sestonic FPOM was filtered from river water over 55 µm Whatmann glass-fiber filters (GF/C), while CPOM was collected in both sestonic and benthic form, by sieving it from the river water and the upper 5 cm of the sediment, respectively. Aquatic macrophytes and epiphyton were both collected with the macroinvertebrate samples.

In the laboratory, the sediment and plant samples were sieved (mesh size 500 µm) and the macroinvertebrates were separated from the sediment and plant material. Macroinvertebrates were then stored at -20˚C until identification. Macroinvertebrates were identified to the lowest practical taxonomic level (generally species).
